# User Engagement Within an Online Peer Support Community (Depression Connect) and Recovery-Related Changes in Empowerment: Longitudinal User Survey

**DOI:** 10.2196/39912

**Published:** 2022-11-02

**Authors:** Dorien Smit, Janna N Vrijsen, Theo Broekman, Bart Groeneweg, Jan Spijker

**Affiliations:** 1 Pro Persona Mental Health Care Pro Persona Research Depression Expertise Centre Nijmegen Netherlands; 2 Behavioural Science Institute Radboud University Nijmegen Nijmegen Netherlands; 3 Department of Psychiatry Donders Institute for Brain, Cognition and Behaviour Radboud University Medical Centre Nijmegen Netherlands; 4 Bureau Bêta Nijmegen Netherlands; 5 Dutch Depression (Patient) Association Amersfoort Netherlands

**Keywords:** depression, online peer support community, internet support group, experiential knowledge, self-management, empowerment, user engagement, longitudinal user survey

## Abstract

**Background:**

The chronic nature of depression and limited availability of evidence-based treatments emphasize the need for complementary recovery-oriented services, such as peer support interventions (PSIs). Peer support is associated with positive effects on clinical and personal recovery from mental illness, but little is known about the processes of engagement that foster change, and studies targeting individuals with depression specifically are limited.

**Objective:**

This study aimed to evaluate whether the level of user engagement, assessed on several dimensions, in an online peer support community for individuals with depression promotes empowerment and the use of self-management strategies and reduces symptom severity and disability.

**Methods:**

In a longitudinal survey conducted from June 2019 to September 2020, we analyzed the data of the users of Depression Connect (DC), an online peer support community hosted by the Dutch Patient Association for Depression and the Pro Persona Mental Health Care institute, on measures of empowerment, self-management, depression, and disability. Of the 301 respondents, 49 (16.3%) respondents completed the survey again after 3 months and 74 (24.6%) respondents, after 6 months. Analysis of 3 parameters (ie, total time spent on the platform, number of page views, and number of posts) derived from their data logs yielded 4 engagement profiles. Linear mixed models were fitted to determine whether the outcomes had significantly changed over time and differed for the various profiles.

**Results:**

Baseline engagement with the online peer support community was “very low” (177/301, 58.8%) or “low” (87/301, 28.9%) for most of the participants, with few showing “medium” (30/301, 9.9%) or “high” engagement patterns (7/301, 2.3%), while user profiles did not differ in demographic and clinical characteristics. Empowerment, self-management, depressive symptoms, and disability improved over time, but none were associated with the intensity or nature of user engagement.

**Conclusions:**

With most DC members showing very low to low engagement and only a few being identified as high-engaged users, it is likely that this flexibility in use frequency is what provides value to online PSI users. In other more formal supportive environments for depression, a certain level of engagement is predetermined either by their organizational or by their societal context; at DC, users can adapt the intensity and nature of their engagement to their current needs on their personal road to recovery. This study added to the current knowledge base on user engagement for PSIs because previous studies targeting depression with an online format focused on active users, precluding passive and flexible engagement. Future studies should explore the content and quality of the interactions in online PSIs to identify optimal user engagement as a function of current, self-reported clinical parameters and reasons to engage in the PSI.

## Introduction

### Peer Support for Recovery in Depression

It is estimated that approximately 280 to 320 million people worldwide are coping with depression [1-3]. However, the availability of evidence-based mental health care, such as psychotherapy and psychopharmacology [4], is insufficient owing to high costs and a lack of skilled clinicians [5]. Moreover, persistent symptoms and high recurrence rates reported underline the chronic nature of the illness [6]. The fact that many individuals live long-term with (recurrent and persistent) depression emphasizes the need for recovery-oriented services that focus on emotional support and resilience rather than on symptom reduction [7]. Peer support interventions (PSIs) could provide such a source of support on the longer road to recovery [8], complementing professional treatment [9,10] for depression [11-13]. In particular, online PSIs meet the need for accessible and low-cost interventions [8], and offering the possibility of anonymous engagement helps circumvent the stigmatization associated with depression [14].

Principally, peer support entails giving and receiving help by exchanging personal experiences [15], where the central themes are “respect, shared responsibility, and mutual agreement of what is helpful” [16]. However, owing to the great variety of intervention types, deployment across different user groups and service delivery settings, there are multiple definitions of peer support [17,18]. Considering this heterogeneity, it is difficult to systematically disentangle the principal benefits of these systems.

### The Effectiveness of Peer Support

We recently conducted a comprehensive meta-analysis of 28 randomized controlled trials (RCTs) to assess the efficacy of PSIs across a wide range of mental disorders and intervention types. Compared with control conditions, the PSIs we reviewed were associated with modest but significant positive effects on clinical symptoms and personal recovery (eg, promoting hope [7]) in individuals with mental illness [19]. Specifically, for individuals with serious mental illnesses, including major depressive disorders, peer support was associated with superior outcomes across clinical, personal, and functional recovery variables (eg, quality of life and social support) relative to control conditions.

It needs to be noted that only a limited number of trials included in our meta-analysis focused on *online* PSIs for *depression*. Nevertheless, the results of few trials were promising. Specifically, findings of the RCT conducted by Griffiths et al [20] suggested that engaging in a moderated depression internet support group may be clinically effective (ie, reducing depressive symptoms) in the long term, with potential short-term improvements for empowerment as presented in a companion paper of Crisp et al [20,21] reporting on the same trial. In addition to this quantitative evidence from a single trial for a depression PSI, descriptive systematic reviews (ie, a narrative synthesis for the efficacy of PSIs, not including a meta-analysis that systematically assesses the results of previously conducted studies) emphasize the potential of online health-related PSIs in general [22-25] and that of those specifically addressing depression [11,26].

The results of a broad systematic review [27] may help us better understand how these positive outcomes in PSIs may develop. Winsper et al [27] identified four common processes fostering change in recovery across 309 studies on recovery-oriented interventions for mental illness: (1) providing information and skills, (2) promoting a working alliance, (3) role modeling for individual recovery, and (4) increasing choice and opportunities. These processes may best be initiated within nonstigmatized, recovery-focused contexts, such as peer support where psychosocial processes of sharing lived experiences, emotional honesty, strengths-focused social and practical support, and the helper-role are important processes for mental health recovery [28]. The results of our qualitative evaluation study for users of the online peer support community Depression Connect (DC) fit these processes (eg, sense of belonging, self-efficacy, and empowerment) [29].

### User Engagement Within Online PSIs

It remains unclear which PSI engagement processes are associated with these changes. In particular, for online PSIs, a high level of user engagement is considered a crucial factor for recovery [24,30,31]. A systematic review of online health communities showed that several multidimensional factors are relevant when defining user engagement, such as metrics characterizing user networks (eg, the number of people a user has interacted with), content (eg, the nature of posts), and activity (eg, the number of posts and log-in times) [32]. Use of online PSIs is mainly operationalized in terms of frequency of use [32], where the dichotomy between “lurkers” (ie, passive users, generally a substantial group, whose use is mainly restricted to reading others’ posts) and “posters” (ie, active users, generally only 1% of users [33-37]) is widely used. To accurately reflect the larger group of passive users, we need a more nuanced characterization of their engagement [32,33]. Such parameters of inactive engagement are particularly relevant for PSIs for depression, as passive behavior is associated with the condition [38]. Therefore, we conducted a *qualitative* evaluation of DC users [29], our self-developed online peer support community for individuals struggling with depression [39]. Our qualitative analysis of user experiences of DC revealed 3 successive participation styles (ie, reading, posting, and responding) that individually and together coincided with an increased sense of belonging, emotional growth, self-efficacy, and empowerment [29]. In this *quantitative* evaluation of DC, we studied engagement patterns as a possible mechanism for recovery more closely by assessing multiple metrics to define engagement as comprehensively as possible [32]. For this study, based on user data logs for DC, we included 3 parameters to operationalize the intensity level of user engagement: the number of posts, the number of page views, and the total time spent on DC. Including both active and passive user modes, it is important to acknowledge that high user engagement was not limited to active users but included users who *posted* (a substantial number of) messages on the platform. In user data logs for DC, it was not possible to distinguish the 2 active participation styles, posting and responding, that is analyzed in our qualitative evaluation. To include passively engaged users in our sample, we assessed *the number of page views* and *the total time spent on DC* per user. However, because users may have been active through sharing posts when viewing various pages on the platform during their time spent on DC, these parameters included, but were not limited to, the passive user mode of reading. Together, our operationalization of user engagement implied that both active users who posted and passive users who spent considerable time on DC and viewed many pages could be categorized as highly engaged users.

### Recovery-Oriented Outcomes

In recent years, peer support studies have frequently reported on personal recovery to complement clinical recovery outcomes, with a particular focus on the benefits of online PSIs for *empowerment* as an important feature in the process of personal recovery that individuals can develop to enable them to live a meaningful life [7,40,41]. Although inconclusive, these findings were promising [21,42-45]. Within online communities, empowerment refers to enabling processes including “becoming better informed, receiving and giving emotional support by sharing relatable experiences of living with the diagnosis, helping others, and networking” [46]. Developing and exploiting self-management strategies can be seen as an active component of empowerment [47,48], and many strategies comprise individual skills “to monitor one’s condition and to affect the cognitive, behavioral, and emotional responses necessary to maintain a satisfactory quality of life” [49]. However, to date, self-management has not been systematically examined as an individual outcome in peer support studies [50]. The same holds true for general well-being (ie, functional recovery, including social functioning and quality of life) [12,42-45,51-53], although both are important parameters for determining the usefulness of recovery-oriented PSIs. Our meta-analysis [19] showed that PSIs may also be effective in terms of clinical recovery (ie, symptom reduction), particularly for individuals with serious mental illness, including major depressive disorder. We also examined whether our online peer support platform helps improve depressive symptoms.

### Objectives

In this longitudinal user survey, we attempted to add to the current literature on online peer support in several ways. First, we examined personal (ie, empowerment and self-management), functional (ie, well-being), and clinical (depressive symptoms) recovery parameters among DC users. Furthermore, we comprehensively explored patterns of user engagement, including parameters reflecting both the intensity and nature of DC use. Following the results of a systematic review of user engagement [32], we clustered user engagement based on the number of posts and page views and total time spent on DC. We aimed to determine whether these user engagement profiles during a 6-month interval were related to changes in empowerment. We expected that user engagement with high frequency—including active (ie, number of posts) and passive (ie, number of page views and total time spent)—would contribute to more improvement in empowerment from baseline to 3 and 6 months compared with user engagement with low frequency. In addition, we explored the use of self-management strategies and changes in depressive symptoms, levels of functioning, and disability over time.

## Methods

### Design

For this longitudinal study, users of our online peer support community for depression, DC, completed an online survey at 3 time points between June 19, 2019, and September 24, 2020.

### Depression Connect

Launched on June 19, 2019, DC was cocreated with experiential experts, caregivers, and health professionals (therapists, psychiatrists, and psychology researchers) affiliated with the Dutch patient association for depression (The Depression Association), the Centre of Expertise for Depression as part of the Pro Persona Institute for mental health care, and the Radboud University Medical Centre. DC was developed to facilitate the exchange of personal experiences in coping with depression among peers. The online platform was easily and (if preferred) anonymously accessible to anyone dealing with depression. Potential users were not screened for depressive symptoms or other clinical characteristics before they could enter the community. Although no professionals were involved in DC, its moderators, who were all experiential experts, were able to consult a psychiatrist and psychology researchers of our team when feedback was needed. To ensure a constructive and supportive online atmosphere, DC moderators screened all new posts daily. They also generated new content or boosted user activity on the platform, for instance, by posting news items or different viewpoints on coping strategies. The DC team welcomed an average of 90 new members each month. DC members could start a new discussion topic or join an existing topic created by other users or provided by the research team. At the DC’s launch, we created 8 forum topics that referred to the main themes of experiential knowledge in depression, which we identified in our qualitative interview study [29]. Widely used topics included coping with symptoms of depression (eg, concentration problems) and treatment options for depression (eg, medication and mental health care). In addition to reading and posting messages on the forum section of the community, users could read news items (posted by the DC team) and read or post blogs. There was also a function for sending private messages to other DC users. More details about the DC’s development, functionalities, and monitoring procedures are presented in our parallel qualitative evaluation of DC [29].

### Participants and Procedure

All individuals who registered with DC were invited to participate in our study when the website was launched. There were no strict conditions to participate in the study regarding demographic and clinical characteristics, either for the minimum or maximum level of engagement with DC. All new DC members, and thus potential study participants, received an email to welcome them to the community, including information about our quantitative evaluation study and a link to the survey. An email address of the research team was also displayed to provide users the opportunity to ask questions about study participation. Participation was on a voluntary basis without any financial or other compensation. Interested users were invited to complete the online survey 1 or 2 days after registering and at 3 and 6 months after joining DC. Of the 1374 new members who joined DC during the recruitment period, 317 (23.07%) users completed the baseline survey. Subsequently, 5% (16/317) of participants deleted their accounts, including their user data logs, leaving 21.91% (301/1374) of participants for the final sample. The data sets of participants who completed only the baseline assessment (179/301, 59.5%) were not included in the outcome analyses.

### Measurements

Participants completed the following measures at baseline and at 3 and 6 months after joining DC.

#### Demographic and Clinical Characteristics

At the baseline assessment, the participants were asked to list their age, sex, and level of education. At all 3 time points, we asked the participants if they received current treatment (referring to any form of mental health care) and used antidepressant medications and whether they were experiencing a depressive episode at the time of the assessment. These variables were assessed by self-report; we did not use a validated symptom-screening measure.

#### User Engagement

Participants’ engagement in DC was determined by analyzing user data logs, which were encrypted and provided by the website host [54]. In line with the most widely used metrics to categorize user engagement in online health communities [32,37], we computed the following three parameters after 3 and 6 months of DC use: (1) total time spent on DC, (2) number of page views, and (3) number of posts entered on DC. We did not consider online activities related to survey completion.

### Outcomes

#### Empowerment

To gauge the changes in empowerment, we used the Netherlands Empowerment List (NEL) [55], that consists of 40 questions covering the following 6 subscales: *social support, professional help, connectedness, confidence and purpose, self-management,* and *caring community*. Items were to be answered on a 5-point Likert scale ranging from 0 (*strongly disagree*) to 4 (*strongly agree*), with a *not applicable* answer option for the *professional help* subscale. We calculated the total empowerment score by summing and averaging all the completed items (range 0-4). Items on the *professional help* subscale that were scored as *not applicable* were not included in this calculation. Higher scores reflected higher levels of empowerment. Both previous research [56] and this study achieved high reliability for the total score (α=.93).

#### Self-management

The use of self-management strategies was evaluated using the Dutch Assessment of Self-management in Anxiety and Depression questionnaire (ASAD) [57,58]. The ASAD considers 45 self-management strategies that are presented in an equal number of statements. Respondents were asked whether and to what extent they used the strategy referred to (eg, *keep focused on the present and stop myself from looking too far ahead*)*.* Each item is rated on a 5-point Likert scale, ranging from 0 (*not at all*) to 4 (*very much*). We used the total score (range 0-180) in our analyses. The higher the score, the higher was the use of self-management strategies. The reliability in this study was high (α=.92). Previous research only examined psychometric properties for the ASAD-Short Form, showing high levels of internal consistency (Cronbach α>.75) for the total questionnaire as well as its subscales (intraclass correlation coefficient >0.75) [46].

#### Depressive Symptoms

Depression severity was assessed using the Dutch version of the Beck Depression Inventory-II (BDI-II) [59], which consists of 21 questions, with each answer scored on a scale from 0 to 3. The total score ranged from 0 to 63, with higher scores reflecting more severe depressive symptoms. Specifically, scores between 0 and 13 indicate minimal symptoms, between 14 and 19 mild depression, between 20 and 28 moderate to severe depression, and the highest category with scores between 29 and 63 indicate severe depression [60]. The BDI-II has good psychometric properties [61]. In this study, the reliability of the total score was high (α=.91).

#### Functioning and Disability

We assessed individual functioning and disability with the Dutch version of the World Health Organization Disability Assessment Schedule (WHODAS) 2.0 [62]. A total of 6 domains (ie *cognition, mobility, self-care, getting along, life activities,* and *participation)* were evaluated with 36 items rated on a 5-point Likert scale, ranging from 0 (*no effort at all*) to 4 (*much effort*), where higher scores indicate more disability. The WHODAS 36 2.0 is a valid and reliable self-report instrument, with good psychometric properties irrespective of population type [63], which was reflected by the high reliability of the total score in our study (α=.92).

### Statistical Analysis

#### Outcomes

Data analyses were conducted using SPSS (version 28; IBM Corp) and R (version 4.1.1) [64] using R Studio (2021.09.0+351). Longitudinal modeling was performed using the R lme4 package [65]. To determine whether the outcomes had significantly changed over time and significantly differed between user engagement profiles, linear mixed models were fitted with the respective outcomes as dependent variables. In the model, the Restricted Maximum Likelihood Estimation calculated parameter estimates. As multiple imputation is not deemed necessary, we did not conduct a missing data analysis a priori [66]. We specified a linear mixed model regression with fixed effects: the actual day of assessment (day), engagement profiles, and the interaction effect between the engagement profile and day of assessment. The baseline value (day=0) of the outcome variable was included as a covariate, and random slopes for the repeated measures design day effect were included.

The estimated marginal means and within-group effect sizes were calculated using the emmeans package [67]. We calculated the magnitude of change between the baseline assessment (day=0) and assessment 3 (day=186), reported as the effect size, Cohen *d* [68]. To calculate the effect size, we needed an estimate of the SD of the intercept. In the model that included the baseline value of the outcome variable as a covariate, the estimate of the intercept’s SD was almost 0. Therefore, we used a model without this covariate to estimate the SD of the intercept.

#### Engagement Profiles

We used cluster analysis to identify subgroups of participants who shared similarities in their forum use patterns. Next, we performed a K-medoids cluster analysis with the cluster package [69], using the partitioning around medoids algorithm, a more robust version of the K-means algorithm, which, instead of averages of distances between points in the sample, uses actual data as the center of a cluster. For each subject, (1) session duration, (2) number of page views, and (3) number of posts were computed for the first 3 months and the last 3 months, excluding the sessions in which the questionnaires were filled out. Because of the extreme skewness in these 6 indices, we took their square roots and transformed them into *z* scores for the cluster algorithm. Although the Tibs2001SEmax gap criterion [69] found an optimum of 7 clusters, the number of participants was very small in the high-engagement clusters (n=3 and n=4), which is why we opted for a 4-cluster solution in which the high-engagement cluster contained 7 (%) participants of the total population (N).

### Ethics Approval

After evaluation, the local ethics committee (Commissie Mensgebonden Onderzoek Arnhem-Nijmegen) determined that no ethics approval was required, given the minimal burden to the study participants. The users provided passive consent to log and analyze their user data.

## Results

### Data Preparation

Of the 301 DC users who had provided their consent and completed the baseline measurement, 179 (59.5%) individuals did not complete the survey at the 3- and 6-month time points. In total, 15.9% (48/301) of DC users completed the 3-month and 24.5% (74/301) of users the 6-month survey. There were no missing data for the 4 main outcome measures at any of the 3 time points. For age and current depression, we noted 2 and 6 missing variables, respectively. A total of 496 observations from 301 participants were included in the mixed modeling analyses.

### Baseline Characteristics of Participants

The participants’ demographic and clinical characteristics as well as the means and SDs for the outcome variables at baseline are shown in Table 1. Our sample of 301 DC users included individuals with self-reported depression and a mean age of 50.2 (SD 13.12) years, 66.1% (199/301) of them were female and most of the respondents (216/301, 71.8%) had completed some form of secondary education or training. More than half of the respondents (166/301, 55.1%) reported having severe depressive symptoms (mean BDI score of 38.7, SD 6.57) and almost one-quarter of the population (72/301, 23.9%) had moderate to severe symptoms (mean BDI score of 23.8, SD 2.53). Of the remaining respondents (63/301, 20.9%), 13% (8/63) reported mild symptoms, and 8% (3/63) had minimal symptoms. The overall mean baseline BDI score for the entire sample was 29.84 (SD 11.85). Most DC users (241/301, 80.1%) received current treatment or some form of support or care from a mental health service and 69.8% (210/301) reported current use of antidepressants.

Completers, that is those respondents that had completed the baseline and at least one second assessment, were on average 4.95 years older (SD 11.4; 2-tailed t_297_=3.25; *P*=.001) and reported significantly higher levels of empowerment, self-management, and less severe depressive symptoms and disability in major life domains compared with DC users who had only completed the baseline assessment.

**Table 1:** Demographic and clinical characteristics of survey respondents at baseline and of the participants having completed at least one subsequent assessment (N=301).

Characteristic			Respondents							Value						
			Total group (N=301)		Baseline only (n=179)		Completers 2 or 3 assessments (n=122)		Test statistic							*P* value
									*t* test (*df*)			*χ*^2^ (*df*)				
Age (years; range 18-99), mean (SD)^a^			50.2 (13.1)		48.22 (13.9)		53.16 (11.4)		3.25 (297)			N/A^b^			<.001	
Female, n (%)			199 (66.1)		119 (66.5)		80 (65.6)		N/A			0.03 (1)			.87	
**Educational level, n (%)**										N/A			11.5 (3)			.01
	None, elementary school, or vocational education	44 (14.6)		36 (12.7)		8 (2.7)										
	Secondary education (middle or high school)	167 (55.5)		90 (29.9)		77 (25.6)										
	Secondary vocational education and training	49 (16.3)		30 (10)		19 (6.3)										
	Advanced vocational education and training and academic education	41 (13.6)		23 (7.6)		18 (6)										
Current depression (self-reported), n (%)^c^			216 (73.2)		136 (63)		80 (37)		N/A			3.7 (1)			.06	
Depressive symptoms (BDI-II^d^), mean (SD)			29.84 (11.9)		31.8 (11.4)		26.97 (12)		−3.53 (299)			N/A			<.001	
**Severity of depressive symptoms (BDI-II)^e^, n (%)**										N/A			11.9 (3)			.008
	Severe depressive symptoms	166 (55.1)		112 (62.6)		54 (44.3)										
	Moderate to severe depressive symptoms	72 (23.9)		39 (21.8)		33 (27)										
	Mild depressive symptoms	39 (13)		19 (10.6)		20 (16.4)										
	Minimal depressive symptoms	24 (8)		9 (5)		15 (12.3)										
Current treatment, n (%)^f^			203 (67.4)		127 (62.6)		76 (37.4)		N/A			2.5 (1)			.12	
Current antidepressant medication, n (%)			210 (69.8)		127 (60.5)		83 (39.5)		N/A			2.9 (1)			.59	
Empowerment (NEL^g^), mean (SD)			2.06 (0.5)		1.99 (0.5)		2.15 (0.5)		2.78 (299)			N/A			.01	
Self-management (ASAD^h^), mean (SD)			78.11 (25.1)		75.15 (26.6)		82.45 (22)		2.5 (299)			N/A			.01	
Functioning and disability (WHODAS 2.0^i^), mean (SD)			35.7 (15.3)		38.11 (15.1)		32.17 (15)		−3.36 (299)			N/A			.001	

^a^Owing to 2 missing variables, n=299 for the total group, n=178 for baseline only, and n=121 for completers.

^b^N/A: not applicable.

^c^Owing to 6 missing variables, n=295 for the total group, n=176 for baseline only, and n=119 for completers.

^d^BDI-II: Beck Depression Inventory-II.

^e^On the basis of the following BDI cutoff scores: 0-13, minimal depression; 14-19, mild depression; 20-28, moderate to severe depression; 29-63, severe depression.

^f^Includes any type of mental health care (eg, general or specialized mental health care and alternative support).

^g^NEL: Netherlands Empowerment List.

^h^ASAD: Dutch Assessment of Self-management in Anxiety and Depression questionnaire.

^i^WHODAS 2.0: World Health Organization Disability Assessment Schedule 2.0.

### User Engagement

Our cluster analysis of the forum uses parameters as described in the Methods section and resulted in 4 user engagement profiles: *very low* (profile 1), *low* (profile 2), *medium* (profile 3), and *high* (profile 4). The user parameters for the total study period (6 months) are listed in Table 2. Baseline engagement profiles did not significantly differ for age; sex; current depression; current treatment or medication; or baseline scores on empowerment, self-management, depressive symptoms, and disability. However, results did show significant differences between participants completing the baseline assessment only and participants that completed one or 2 assessments for the “very low” engagement profile (177/301, 58.8%), of which 33.9% (60/177) participated in a second assessment and 66.1% (117/177) completed baseline only (χ^2^_3_=27.1; *P*<.001; Multimedia Appendix 1).

**Figure 1:**
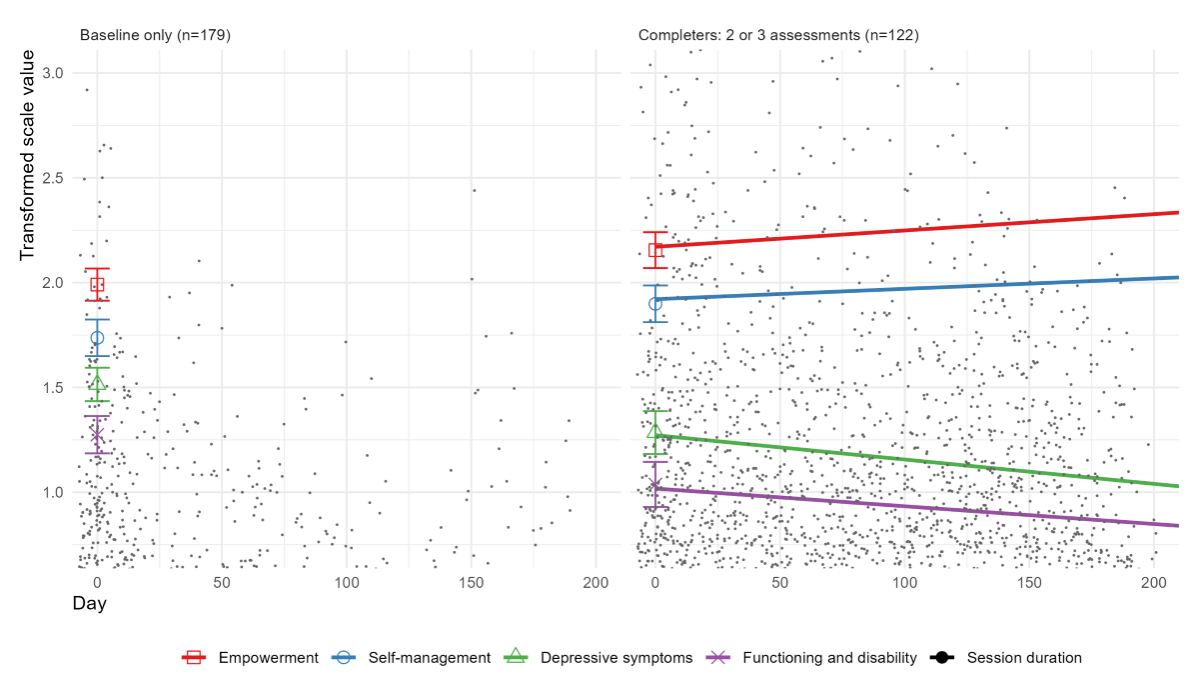
Session duration and changes in empowerment, self-management, depressive symptoms, and functioning and disability over 6 months of using Depression Connect.

Figure 1 depicts the (changes in) outcomes and session duration for the participants who completed only the baseline assessment (panel 1, n=179) and for those who completed at least 2 or all 3 assessments (panel 2, n=122). The graphs present data modeled using a longitudinal mixed model regression analysis for session duration for each individual session (black dots), empowerment (NEL), self-management (ASAD), depressive symptoms (BDI-II), and functioning and disability (WHODAS 2.0) over time, including the baseline means for each outcome. They show an increase in empowerment and self-management and a decrease in depressive symptoms and disability over time (days of engagement with the online peer support community—DC).

In the figure, the scale values were transformed to a range from 0 to 4. To avoid overlap, self-management was raised by 0.4 and functioning and disability was lowered by 0.25. The session duration is transformed to the square root of the duration in minutes and divided by 5, so that most values are in the figure. These lines are based on the values predicted by the model. Values at day 0 are actual means with the confidence levels.

**Table 2:** Depression Connect user engagement for the total study group (N=301).

Total study group (engagement parameter)	Very low engagement (profile 1; n=177), mean (SD)	Low engagement (profile 2; n=87), mean (SD)	Medium engagement (profile 3; n=30), mean (SD)	High engagement (profile 4; n=7), mean (SD)	Value, mean (SD)	*F* test (*df*)	*P* value
Total session duration in hours	5.48 (22.37)	0.37 (1.1)	2.35 (2.43)	18.84 (17.04)	116.01 (86.20)	177.51 (3,297)	<.001
Number of page views	322.19 (1117.14)	25.85 (41.71)	161.18 (103.12)	1315.33 (1015.74)	5560 (4159.86)	175.63 (3,297)	<.001
Number of posts	14.21 (58.45)	0.33 (1.33)	5.79 (5.81)	44.8 (47.46)	338.7 (158.63)	363.26 (3,297)	<.001

### Outcomes

Table 3 lists the results of the models that investigated changes in outcomes over time (days of DC use). We computed a significant increase in empowerment (NEL) over time in days (β=.00078; SE 0.00022; *P*=.001) with a small effect size (Cohen *d*=0.36, 95% CI 0.15-0.57). Self-management also increased over time (β=.0222; SE 0.011; *P*=.046), again with a small effect size (Cohen *d*=0.22, 95% CI 0-0.43). Depressive symptoms (BDI) significantly decreased over time with a small effect size (β=−0.0244, SE 0.00612; *P*<.001; Cohen *d*=0.44, 95% CI 0.21-0.66). In addition, disability (WHODAS 2.0) significantly decreased over time with a small effect size (β=−0.0212, SE 0.00693; *P*=.001; Cohen *d*=0.29, 95% CI 0.10-0.47).

Engagement profiles were not significantly associated with changes in any of the outcomes at 3 or 6 months as indicated by the nonsignificant effects of the dependent variable by engagement profile: empowerment (*F*_3,176_=0.07; *P*=.98); self-management (*F*_3,169_=0.1; *P*=.96); depressive symptoms (*F*_3,184_=0.14; *P*=.94); and functioning and disability (*F*_3,181_=0.2; *P*=.90); and nonsignificant time by profile interactions: (empowerment (*F*_3,131_=0.55; *P*=.65); self-management (*F*_3,126_=.93; *P*=.43); depressive symptoms (*F*_3,140_=0.09; *P*=.97) and functioning and disability (*F*_3,158_=0.09; *P*=.96).

**Table 3:** Linear mixed model analysis outcomes; estimated marginal means and effect sizes^a^.

Outcomes	Baseline, EMM^b^ (SE)	3 months (day 95), EMM (SE)	6 months (day 186), EMM (SE)	Fixed effect of time				Cohen *d* effect size^c^ day 0 to day 186 (95% CI)
				Change, per 100 days	*F* test (*df*)	*P* value		
NEL^d^ (empowerment)^e^	2.17 (0.01)	2.24 (0.02)	2.32 (0.04)	.078	12.5 (1,136)	.001	0.36 (0.15-0.57)	
ASAD^f^, self-management^g^	83.44 (0.7)	85.56 (0.98)	87.58 (1.89)	2.22	4.05 (1,132)	.046	0.22 (0.0-0.43)	
BDI-II^h^ (depressive symptoms)^i^	26.71 (0.36)	24.4 (0.55)	22.18 (1.04)	−2.44	15.9 (1,147)	<.001	0.44 (0.21-0.66)	
WHODAS-36 items 2.0^j^ (disability)^k^	31.69 (0.53)	29.68 (0.60)	27.76 (1.11)	−2.12	9.33 (1,164)	.003	0.29 (0.10-0.47)	

^a^We used a linear mixed model with time (days) and baseline value of the dependent variable as a fixed factor and subject within time (days) as random effects.

^b^EMM: estimated marginal mean.

^c^To calculate effect sizes, we used a model in which the baseline value of the outcome variable as a covariate was not included because in the model including this covariate, the estimate of the intercept’s SD naturally almost 0.

^d^NEL: Netherlands Empowerment List.

^e^Scores range from 0 to 4, with higher scores indicating greater empowerment.

^f^ASAD: Dutch Assessment of Self-management in Anxiety and Depression questionnaire.

^g^Scores range from 0 to 180, with higher scores indicating higher use of self-management strategies.

^h^BDI-II: Beck Depression Inventory-II.

^i^Scores range from 0 to 63, with higher scores indicating more (severe) symptoms.

^j^WHODAS 2.0: World Health Organization Disability Assessment Schedule 2.0.

^k^Scores range from 0 to 144, with higher scores indicating greater disability.

## Discussion

### Principal Findings

Although the potential benefits of engaging peer support for people with severe mental illness (SMI) are widely acknowledged [25,26], peer support studies are limited for online intervention types targeting depression [20,21] and the processes for user engagement remain unclear [32,70]. In this longitudinal user survey of the online peer support community—DC, we explored patterns of user engagement and examined whether user profiles were associated with recovery-oriented outcomes. To quantify baseline to 6-month changes in empowerment, self-management, depressive symptoms, and functioning and disability in the users of DC and considering the complex interplay of relevant aspects of user engagement in PSIs, we entered the user data logs of 3 parameters (ie, total session duration, page views, and number of posts) into a cluster analysis, resulting in 4 engagement profiles. Most of the survey respondents (177/301, 58.8%) had very low or low engagement levels (87/301, 28.9%), with 9.9% (30/301) having medium and 2.3% (7/301) high user profiles. However, none of the profiles showed significant differences for age; sex; having current depression; receiving treatment at the time of assessment; or with regard to the baseline scores for empowerment, self-management, depression, and functioning and disability. All recovery-oriented outcomes improved over time; however, contrary to our hypothesis, the nature and intensity of DC user engagement were not significantly associated with any of these improvements.

### Findings in Context

The number of user surveys and RCTs for online PSIs for depression is limited; however, the results are promising. Although our results did not show a significant relationship between the level of user engagement and recovery, Griffiths et al [20,21] reported positive results for engaging a online PSI for depression in their trial. They found that depressive symptoms reduced in the long-term period (6 and 12 months) and empowerment may improve in the short-term (after the intervention or at 3 months). Furthermore, reviews with and without meta-analyses of PSIs that include a heterogeneous population, which includes primarily individuals with SMI, report positive changes in psychosocial outcomes [24,25,43,45], more specifically for self-efficacy and hope [12,42,44,51-53]. We confirmed this in our new and updated meta-analysis, which included PSIs for mental illness [19]*.* However, research on peer support is associated with methodological issues (eg, establishing model fidelity is not possible at this point [25]). Therefore, the results of this longitudinal user survey as well as the results from the other PSI studies should be interpreted with caution.

Considering the level of user engagement, it is generally known that online communities [37,70] are associated with low engagement rates. This is often referred to as the 1% rule [36,37] and is in line with our results. However, our study adds to the current literature, as the results improve insight into the intensity and nature of user engagement for online PSIs for depression. Further research is needed to better understand the relationship between levels of user engagement and positive changes in recovery.

### Flexible User Engagement

#### In Search of a Valid Proxy Measure for the Nature of Forum Use

To create as true a proxy as possible for the way the participants in our study used the DC platform, we included multiple indicators that we thought would best reflect the nature of their forum use. However, our results surprisingly showed that the frequency and nature (passive vs active) of user engagement did not appear to be associated with recovery. We focused on presence and participation rates on DC, whereas the CAPE model states that a broad range of factors should be incorporated when operationalizing user engagement. CAPE is an acronym including metrics on the following factors: Connect (how many people are interested), Attend (eg, presence or how many log-ins), Participate (eg, active engagement), and Enact (making use of online learned skills in daily life) [70,71]. Adding to the current knowledge base on user engagement, our results suggested that it might be too simplistic to assume that there is an optimal or specific engagement pattern or style that is directly related to positive outcomes associated with the use of PSIs [32]. As self-determination is a crucial aspect of the recovery-oriented approach, which is reflected in our PSI, voluntary use of the program seems important [72]. Arguably, the need for support from peers or the intention to support peers depends on the stage of depression or coping levels, which affects the intensity (ie, frequency or duration) and nature of a person’s forum engagement (eg, posting to ask for help or responding to help others) [73]. In line with our qualitative evaluation of DC user experiences, the data presented here might indicate that user modes are indeed used interchangeably over time, developing and deploying different engagement styles (ie, reading, posting, or responding) according to their personal needs [29]. Therefore, these shifts in forum use make it difficult to capture the effects of DC use in quantitative terms, such as engagement profiles. Nevertheless, online PSIs appear to provide users with an accessible digital realm in which they are free to choose individual modes of engagement that match their current needs in their search for recovery.

#### Quantity Versus Quality of User Engagement

In addition, the perceived quality of forum posts might be a relevant factor to be included when defining user engagement in terms of nature and intensity. It is possible that low engagement with the DC community suffices to benefit from peer support if a recently published post answers a specific question or explores a relevant topic effectively, satisfying the current needs of individual users [74]. DC users with queries about treatment options, for instance, may not have needed to spend much time on the platform to find pertinent information or check whether they had received a fitting response. In turn, if a user is looking for (online) friendship (to create a sense of belonging), they are likely to spend more time on the forum and engage more actively to connect with peers. Taken together, it may well be the personal needs and goals of the users and the perceived quality of the forum content that ultimately determine whether and how users engage in and benefit from online peer support communities, such as DC.

#### Potential Disadvantages of Active User Engagement

From a different perspective, 2 potential disadvantages of active forum engagement might have defeated the hypothesized positive association between high user engagement and the experienced benefits (recovery indices) of DC. First, the data showed that high-frequency users (high engagement) posted significantly more messages than the users with the other 3 profiles (very low, low, and medium engagement). This might imply that frequent users predominantly posted messages for (ie, responded to) peers seeking support, focusing less on their own needs and recovery. According to the helper-therapy principle [75], high-frequency users may experience positive feelings because they perceive helping peers as meaningful. In line with the central drawback that DC users emphasized in our qualitative study, this active style may also have increased distress levels by their feeling responsible for their peers’ well-being or by their identification with the problems of fellow users too much [17]. In addition, as observed in clinical practice [76] and our qualitative study [29], high engagement in supportive interactions may encourage self-reflection, uncovering problems that users were not, or partly, aware of before, which might be both distressing and healing. Thus, compared with passive users, active engagers run a greater risk of being exposed to the disadvantages of peer support, possibly increasing their disease burden owing to a heightened sense of responsibility for others and an increased awareness of their personal issues.

### Assessing Recovery in Online Peer Support

Finally, other recovery-oriented outcomes may be more relevant for evaluating an unstructured online peer support community such as DC. Empowerment and self-management may serve as those attributes that would characterize more advanced stages of recovery from mental illness, such as depression, as they take time to develop and generally require guidance from a nonpeer (ie, a paraprofessional) [18,46], face-to-face PSI format [42,43,77], or a wider supportive context involving family or friends [78,79]. Moreover, considering the informal nature and flexibility, free use of our platform, and the fact that our sample mostly consisted of individuals with moderate to severe depressive symptoms, smaller goals such as an increased sense of being (emotionally) supported or finding new hope are probably more feasible [80].

### Limitations

This study has several limitations. The first one lies in the operationalization of user engagement. Rather than opting for (more frequently used) self-report measures, we tried to objectively quantify forum engagement using logged user data (total time spent on the platform and the number of page views and posts); however, there are other potentially relevant indicators of engagement, such as the number of posts the user reads [34], the length of threads [74], and the number of replies received [74,81]. Particularly for individuals with depression, these activities and interactions that reflect recognition and support may reduce stress and negative emotions [82]. Unfortunately, we were unable to extract these parameters from the user data logs. Second, the results of our previous qualitative exploration of DC user experiences were primarily related to users with an active engagement style. In this quantitative study, the number of highly frequent and actively engaged users—those posting significantly more than their peers with other engagement profiles—was too small to detect any reliable effects on empowerment. Third, the lack of a comparison group in this longitudinal user survey precluded exploration of causal relationships between DC use and recovery; however, the effect sizes of RCTs comparing PSIs for mental illness with a control group that we pooled in our meta-analysis were significant both for clinical and personal recovery indices.

Finally, the generalizability of our findings is limited as we evaluated self-selected samples, where the decision to participate may contain some inherent positive bias toward engaging in (online) peer support. It is possible that users with a low engagement profile were not motivated to complete the follow-up assessments in our evaluation study because they lacked commitment to DC or may not have experienced any benefits of engagement. However, our study has an explorative character, with a naturalistic sample that informed us of the general and heterogeneous population of individuals with depression who engage in peer support. Given the observational character of our study, its internal validity is limited. We do not know whether the improved outcomes are related to DC use and to what extent other types of support or the many other variables that are part of the real-world setting (eg, the level of offline social support, self-stigma, and societal participation) influence these results. Regardless, considering the free and informal nature of our online peer-to-peer support environment that allows users to tune their use of the forum to their personal needs and the improvements observed, our survey expands the current literature by focusing on an online PSI for depression. The results underscore that this type of peer support appears to be beneficial and promotes recovery among individuals with self-reported depressive symptoms. These promising results are not only reflected in our survey but also in previously conducted user surveys in PSIs, underscoring the benefits of peer support for clinical [14] and personal recovery [33].

### Future Research

As the various engagement profiles that we identified indicate that DC users appear to prefer a flexible use of the platform, insight into the content of their posts would foster the interpretation of our findings. Therefore, we recommend assessing the perceived quality of interactions (eg, “Is the content helping you to cope with your depression?”) in future research on online PSIs. The quantitative variables such as thread length and the number of posts and responses or comments might indicate how effectively a topic was explored [74]. Synthesizing qualitative data (content analysis) and quantitative data (metrics of use) of peer support user engagement would enhance our understanding of its implications for recovery. In addition to the clinical characteristics and (treatment) history of depression, it may be informative to describe the societal context of individual users. Possibly, the availability and quality of social support from family or friends may predict users’ need for online peer support and explain their low or high engagement. As peer support is considered adjunctive to formal mental health care [9] and it has been suggested that peer support encourages users to engage more actively in their professional treatment [14], it is worthwhile to investigate the usefulness and benefits of (online) peer support for concurrent professional therapy. Finally, recovery is a multidimensional concept; however, the various factors and processes involved are difficult to disentangle. Including comprehensive measurements in which the umbrella concepts of clinical, personal, and functional recovery-related indices are assessed separately and in depth, such as in the case of the Recovery Assessment Scale, might improve the validity of the findings.

### Conclusions

This longitudinal user survey provides insights into the characteristics of user engagement in DC, an online peer support community for depression. Active engagement was limited to a small group of DC users and was not significantly associated with superior improvements in empowerment and secondary recovery-oriented outcomes. Users appear to attune the intensity and nature of their forum use to their personal recovery pathway and current needs, where their engagement levels may shift from low to high and from passive to active. Corresponding to the self-determination theory, the autonomy to choose the level of engagement might be one of the most valued and effective features of intervention types, such as DC, whereas in other more formal supportive environments for depression, a certain level of engagement is predetermined. Future online PSI studies should explore the content and quality of user interactions to determine what constitutes optimal user engagement, where flexibility and usefulness match users’ clinical needs and motives to seek and offer online peer support.

## Appendix

Multimedia Appendix 1 Baseline demographics and clinical characteristics for the total study group.

## Abbreviations

ASAD: Dutch Assessment of Self-management in Anxiety and Depression questionnaire
BDI-II: Beck Depression Inventory-II
DC: Depression Connect
NEL: Netherlands Empowerment List
PSI: peer support intervention
RCT: randomized controlled trial
WHODAS: World Health Organization Disability Assessment Schedule 2.0

## Data Availability and Management

All data and data management were handled in accordance with the national guidelines for university faculties of social and behavioral sciences (formulated by the DSW Committee: Deans of social sciences faculties, March 2022 [83]). Accordingly, to enhance open science and transparent research practices, we followed the FAIR principles (findable, accessible, interoperable, reusable) and stored (descriptions of) our study material and approaches as open as possible and as closed as necessary at the Radboud Data Repository.
