# Supplementary material for: User Engagement Within an Online Peer Support Community (Depression Connect) and Recovery-Related Changes in Empowerment: Longitudinal User Survey
Source: JMIR Form Res. 2022 Nov 2;6(11):e39912. doi: 10.2196/39912 (PMC9669893; doi:10.2196/39912)
Supplement: Multimedia Appendix 1 [file formative_v6i11e39912_app1.docx]

**Multimedia Appendix 1.**

Baseline demographics and clinical characteristics for the total study group

|  | **Total study group (N=301)** | **Very low engagement (profile 1) (n=177)** | **Low engagement**  **(profile 2) (n=87)** | **Medium engagement (profile 3) (n=30)** | **High engagement (profile 4) (n=7)** | **Test statistic** | ***P value*** |
| --- | --- | --- | --- | --- | --- | --- | --- |
|  | *Mean (SD) or frequency (%)* | *Mean (SD) or frequency (%)* | *Mean (SD) or frequency (%)* | *Mean (SD) or frequency (%)* | *Mean (SD) or frequency (%)* | *F(df) or χ²(df)* |  |
|  |  |  |  |  |  |  |  |
| **Characteristic** |  |  |  |  |  |  |  |
| Age in years (range 18-99), mean (SD)^a^ | 50.2 (13.12) | 50.49 (13.15) | 48.33 (13.35) | 52.42 (13) | 57.33 (5.41) | F(3,295) = 1.59 | .19 |
| Female, n (%) | 199 (66.1) | 114 (57.3) | 63 (31.7) | 18 (9) | 4 (2) | χ²(3) = 2.5 | .47 |
| Current depression (self-reported), n (%)^b^ | 216 (73.2)^b^ | 123 (56.9) | 69 (31.9) | 21 (9.7) | 3 (1.4) | χ²(3) = 5.1 | .16 |
| Current treatment, n (%)^c^ | 203 (67.4) | 118 (58.1) | 61 (30) | 22 (10.8) | 2 (1) | χ²(3) = 0.6 | .13 |
| Current antidepressant medication, n (%) | 210 (69.8) | 130 (61.9) | 59 (28.1) | 17 (8.1) | 4 (1.9) | χ²(3) = 4.3 | .23 |
| Empowerment (NEL), mean (SD) at baseline | 2.06 (.51) | 2.04 (0.5) | 2.07 (0.55) | 2.09 (0.45) | 2.23 (0.32) | F(3,297) = 0.42 | .74 |
| Self-management (ASAD), mean (SD) at baseline | 78.11 (25.07) | 78.87 (25.1) | 75.59 (25.21) | 80.6 (25.57) | 79.57 (23.41) | F(3,297) = 0.45 | .72 |
| Functioning and Disability (WHODAS 2.0), mean (SD) at baseline | 35.7 (15.3) | 36.49 (15.79) | 34.73 (15.29) | 34.04 (13.13) | 35.17 (13.03) | F(3,297) = 0.39 | .76 |
| Depressive symptoms (BDI-II), mean (SD) at baseline | 29.84 (11.85) | 30.24 (12.5) | 29.34 (11.24) | 30.07 (10.71) | 25 (6.43) | F(3,297) = 0.51 | .68 |
| Completers (2 or 3 assessments), n (%) | 122 (40.5) | 60 (34%) | 33 (37.9) | 22 (73.3) | 7 (100) | χ²(3) = 27.14 | <.001 |

Abbreviations: BDI, Beck Depression Inventory; M, Mean; NEL, Netherlands Empowerment List; SD, Standard deviation; SM, Self-management skills questionnaire; WHODAS, World Health Organization Disability Assessment Schedule.
^a^Due to 2 missing variables, n=299 for the total group, n=176 for profile 1; n=86 for profile 2; n=30 for profile 3; n=7 for profile 4 ^b^Due to 6 missing variables, n=295 for the total group, n=174 for profile 1; n=85 for profile 2; n=30 for profile 3; n=6 for profile 4
^c^Includes any mental health care (eg, general or specialized mental health care, and alternative support)
